# On the Practical Study of Botany, by Means of Chemical
Analysis:—Chiefly Abridged from the Original Papers of Dr. F. S.
Hermbstaedt, &c.

**Published:** 1799-04

**Authors:** 


### *On the practical Study of Botany, by Means of Chemical Analyſis:—chiefly abridged from the original Papers of* Dr. F. S. Hermbstaedt, &c.

[*Continued from Number I. p.* 72—77.]

### TABLE SECOND.

*A general View of the* SECONDARY ELEMENTS *of vegetable Bodies, which cannot be exhibited as Objects of Senſe, in a ſeparate State.*

1. THE *aſtringent* principle.—Subſtances which are ſuppoſed to contract the animal ſolids (or according to others, to produce ſome beneficial change in the body, either by ſtimulating the nerves of the ſtomach, or affecting the maſs of the fluids), are, in a *medicinal ſenſe*, termed *aſtringents.* This definition is, however, very imperfect; inaſmuch as by aſtringents are more properly meant, *tonics*, or ſuch ſtrengthening remedies as are given for the purpoſe of bracing the elementary fibres of the body, when they are perhaps too looſely connected, and of relieving weakneſs in the ſyſtem, ariſing

---

arising, as is conjectured, from want of *tone*, or defect of elasticity in the vessels *.

In a *chemical sense*, which more properly applies to the subject of the present inquiry, the *astringent* principle, in general, denotes only that property inherent in vegetable substances, by which leather is tanned, and a black colour produced, when combined with solutions of iron. This astringent property is most copiously contained in *galls*, and is in every respect analogous to that contained in the Peruvian bark, and many other astringent and bitter vegetables.—The *gallic acid of* SCHEELE is not a *pure* astringent substance, but is combined with a considerable portion of vegetable acid. Its presence may be easily discovered, by making the experiment with a saturated solution of iron, which thus exhibits a black precipitate. This astringent principle is rendered volatile in a warm temperature, without losing the property of striking a black colour.

2. The *colouring* principle. This is unquestionably connected with a peculiar modification of astringency, as all colouring vegetable substances possess more or less of that property. The origin and phenomena of the different colours, [are probably founded on the different degrees of refraction of the rays of light proceeding from the coloured object towards the eye.

3. The *acrid*, or pungent principle. This is of a volatile nature, and may be totally expelled by heat. It is most copiously found in horse-radish, mustard, onions, &c.; but it cannot by any means be exhibited completely pure and free from other ingredients. The very pungent or burning quality of the *fresh* root of the wake-robin (*Arum maculatum. Linn.*), the pellitory of Spain (*Anthenis pyrethrum, Linn.*), especially in a *dry* state; the mezereon †, *Daphne*

* As the want of cohesion, so frequently observed in the fibres of the human body, is probably owing either to a watery consistence, or diminished proportion of the animal gluten in the circulating fluids, it is evident, that proper articles of food, such as contain this jelly in considerable proportion, afford likewise the most effectual means of improving the fluids, restoring the losses sustained, and thus invigorating the whole body. This, however, is a theory much contested, as being the grand pillar of what is now, by many, called in derision, '*humoral pathology*'. Without deciding on the merits of *humoral*, *nervous*, or (what is the most absurd of any,) CHEMICAL PATHOLOGY, we shall only remark, on this occasion, that it is of the utmost consequence to the medical practitioner, to determine with critical precision, whether the diminished action, as well as the want of cohesion in the fibres, proceed from the impaired state of the fluids, and particularly the gluten, or from too great extension and subsequent relaxation of the fibres themselves. W.

† The *mezereon* and *anemone* have lately been introduced into medicine, on account of their active and remarkable effects on the human system, in cases of lues, scrofula, &c.; but I cannot forbear remarking, with indignation, that the ever-busy London quacks

(*Daphne Mezereum, Linn.*); the meadow anemone, (*Anemone pratenſis Lin.*) and ſeveral other vegetables, is entirely owing to this acrid quality.

4. The *narcotic* principle is contained chiefly in thoſe vegetables which, if uſed in ſufficient quantity, have a direct tendency to induce ſleep, ſuch as the poppy, the hemlock, and the deadly night-ſhade. It is of a volatile nature, as is evident from the pernicious exhalations of narcotic ſubſtances, when they are expoſed to the action of heat. But this deleterious principle is ſo intimately combined with the baſis of gum and ſoap, that it cannot be exhibited in a ſeparate elementary ſtate.

5. The *bitter* principle. It has not yet been determined with accuracy, whether a peculiar bitter ſubſtance really exiſts in nature, or whether the bitter taſte of many vegetable bodies is the reſult of their combination with other conſtituent parts. Nor has any ſubſtance whatever, hitherto, afforded this principle in a free or uncombined ſtate; for, in the chemical analyſis of vegetable bodies, we always find it blended with gum, mucilage, or ſoap.

6. The *odoriferous* principle appears to be moſt intimately combined with the eſſential oils of vegetables, expreſſed or diſtilled. It has been already remarked, that it is not yet decided by ſufficient experiments, whether the odoriferous principle is not reſiding in ſome other element, totally different from that of the oil; as many vegetable bodies emit remarkable fragrance without affording any quantity of eſſential or diſtilled oil, in proportion to the degree of their odour*.

TABLE

quacks have already seized upon these powerful substances, in order to make them the *ostensible* agents in their "*Botanical Syrups, Vegetable Balsams,*" &c; when, in fact, the principal ingredients of their nostrums, consist of the most virulent metallic preparations, such as sugar of lead, the oxydes of copper, mercury, arsenic. &c.

W.

* We cannot, in this instance, reason from analogy; for some vegetables apparently abounding in oil, yield very little of it, and others none at all; for instance, roses and chamomile flowers, though of a strong and permanent smell, yield but a small quantity of oil; a pound of the former producing no more than from three to four grains of essential oil, and a pound of the latter, about half a drachm, or two scruples. The violet and jessamine-flowers, which perfume the air with their odour, are deprived of this agreeable property, by exposing them to a very moderate fire, and do not afford the least particle of oil in distillation, unless immense quantities be at once submitted to this process; while savin, the unpleasant scent of which is perceived at a considerable distance, parts with the largest proportion of oil of almost any known vegetable; insomuch that one pound of it yields from two to three ounces of essential oil, being the eighth, and sometimes the sixth part of the weight of the plant.

TABLE THIRD.

*Simple Vegetable Acids.*

1. The acid of *tartar*, or tartarous, acid, which is obtained from ſome vegetable ſubſtances, hereafter to be ſpecified, in a pure ſtate, while in others we find it combined with alkaline ſalts and earths;—it is frequently blended with other vegetable acids, as well as with the matters of gum and ſoap. This acid forms rhomboidal cryſtals, which ſwell when expoſed to the action of a moderate fire, emit the ſmell of burnt cream of tartar, and at laſt leave behind a ſpongy coal in the crucible.

2. The acid of *apples*, or the *malic* acid, is moſt copiouſly contained in the juice, both of ripe and unripe apples. SCHEELE was certainly the firſt chemiſt, who taught us to ſeparate this acid, which ſo far differs from the acid of tartar, that it does not form cryſtals, and, if combined with calcareous earth, is more eaſily ſoluble than the tartarous acid.

3. The acid of *lemons*, or the *citric acid*, is combined with the malic acid in the juice of lemons, in the ſap of gooſberries, as well as in a variety of other fruits, but particularly in the barberry or pipperidge-fruit. It forms lamellated cryſtals, which poſſeſs a more acrid taſte than thoſe of the tartareous acid, and which, when combined with magneſia, are eaſily ſoluble in water.

4. The acid of *ſorrel*, or the *oxalic* acid (formerly called acid of ſugar, or ſaccharine acid), cryſtallizes in rhomboidal columns, and is readily exſiccated, or reduced to powder, if expoſed to the action of atmoſpheric air. Its cryſtals diſſolve in water with a peculiar ruſtling noiſe, and poſſeſs an acrid ſour taſte.

The acid of *benzoine*, or the benzoic acid, is ſufficiently known by the name of *flowers of benzoine*. This ſubſtance apparently conſiſts of a baſis rather compounded than ſimple. Beſides the concrete reſinous juice of benzoine, this acid is alſo diſcoverable in the Peruvian balſam, in the watery infuſion made of the flowers of cinnamon and caſſia, and in a few other vegetables.

6. The acid of *milk*, or the *ſaccho-lactic* acid, is produced from the ſugar of milk, by boiling it with a proportionate quantity of nitric acid. It may likewiſe be obtained from gum arabic, and the mucilage of tragacanth. This acid appears to be a conſtituent part of many vegetables, and, on that account, deſerves great attention in their chemical analyſis.

There are ſeveral other vegetable acids uſually enumerated by authors, ſuch as the *gallic* acid, which has been ſtated in the SECOND TABLE, as forming the *aſtringent principle;* the *pyro-ligneous* acid, formerly termed empyreumatic acid ſpirit of wood; the *pyro-mucous* acid, otherwiſe called ſyrupous acid, or or ſpirit of ſugar, honey &c.—the *pyro-tararous*, or ſpirit of tartar; the *ſuccinic*, commonly called acid of amber; and perhaps a few others: but all theſe now ſpecified, are either modifications of one or other of the preceding ſix ſpecies, or they cannot be ſtrictly called *vegetable acids;* for inſtance the *ſuccinic*, or the acid of amber, which is evidently obtained from a bituminous ſubſtance, though its vegetable origin can hardly be diſputed.

In the preceding three Tables, we have merely enunciated the primary and ſecondary conſtituents of vegetable bodies, without attending to the artificial products, to be obtained from them, or to their reſpective effects on the human body. Before, however, we can attempt to enter upon the diſcuſſion of theſe important objects, it will be uſeful and neceſſary to premiſe a few particulars relative to the *laws*, by which a chemical analyſis of vegetable bodies muſt be conducted, and the views with which it ought to be undertaken.

Although *ſuch* an analyſis ſhould not be productive of *immediate* and *extenſive* benefit to the chemiſt, whether conſidered as a man of ſcience, or a commercial operator, yet it will be admitted, that it is of the utmoſt conſequence, and promiſes great and indubitable advantages to the medical practitioner.

When we endeavour to inſtitute an accurate analyſis of vegetable bodies, we ſhould never loſe ſight of this fundamental propoſition, *that every plant is a perfect organic body.* We ought, farther, to direct our conſtant attention to the following axioms: 1. That organic bodies, in a temparature conſiderably above the boiling point of water (212° of Fahrenheit, or 80° of De Luc), are not decompoſed in ſuch a manner as to exhibit their proximate, but rather their remote conſtituent parts; and 2. That in the re-union of the latter, in different proportions to each other, new chemical products reſult from the operation;—products which, in this ſtate, formed no elementary principle in the vegetable body. If theſe circumſtances be duly conſidered, no further proof will be demanded, that the proceſs of analyſing ſuch ſubſtances in the *dry way*, (as hitherto practiſed by all the ancient and many modern operators, with whom the agency of fire, and the uſe of an alembic, were the principal and almoſt only requiſites to a chemical laboratory) is in every reſpect inſufficient to diſcover the true elementary conſtituents of bodies ſubject to ſuch analyzation.

According to theſe premiſes, the temperature in which the chemical analyſis of organic ſubſtances is to be performed with ſucceſs, muſt never exceed the boiling point of pure water. In order to aſcertain this point with a ſufficient degree of accuracy (eſpecially as the co-operation of heat is in almoſt every every inſtance abſolutely neceſſary to effect the decompoſition of organic bodies), a vehicle ought to be employed, which is capable of checking any exceſs of the temperature before ſtated.

Such a vehicle we find in the pureſt water. To this ſubſtance, if its expanſion be not prevented, by confining it in cloſe veſſels, and excluding the acceſs of atmoſpherical air, we can eaſily communicate the greateſt quantity of free *caloric* (matter of heat), without exceeding, in any remarkable degree, the maximum of the temperature it has thus acquired;—provided however, that during the experiment there be no greater variation in the ſtate of the barometer, than from 29 to 30 inches.

Although the truth of this propoſition is now fully eſtabliſhed, it will neverthelefs be uſeful, and even neceſſary, for the information of thoſe who are not thoroughly acquainted with the particulars of the experiments made on this curious ſubject, to premiſe a ſhort analytical account of the facts on which it is founded, and by which it is ſupported:—

If a pound of ice or ſnow, the natural temperature of which is uniformly 32° of Fahrenheit, be mixed with a pound of water, heated to 172° of the ſame ſcale, it will be found that the mercury in the thermometer plunged into this mixture, even after all the ice or ſnow has been melted, does not riſe higher than 32°—ſo that the temperature of the mixture remains perfectly equal to that which the ice or ſnow poſſeſſed, previous to the addition of hot water: as the temperature of this, which before was 172°, is thus deprived of 140° of its heat, and reduced to 32°, or the freezing point, the deficient 140° of caloric have combined with the ice; and although they have not in the leaſt affected its temperature, they have nevertheleſs changed its form, and converted it from a concrete into a fluid body. On the ſtrength of this experiment, the following aphoriſm is incontrovertibly eſtabliſhed: *That caloric may unite with bodies in conſiderable quantity, without producing any change in their temperature; but their forms are, in this combination, neceſſarily changed.*

If we farther apply this invariable law of phyſics, in communicating free caloric to water itſelf, the temperature of which is 32°—if a quantity of free caloric be imparted to it, till it acquire the higheſt degree of heat it is ſuſceptible of, namely that of 212°, we ſhall then find, that a new relation takes place between the water and the caloric. For, if we attempt to communicate a ſtill greater quantity of caloric to the water already heated to 212°, it is no longer ſuſceptible of additional heat, but the caloric enters into a new chemical combination with the water. And if the ſtate of the barometer remain as before ſtated at 30°, and that of the thermometer at at 212°, a new phenomenon will preſent itſelf to the obſerver: the water will change its form a ſecond time; and it will be converted from the fluid ſtate to that of vapour—in conſequence of the caloric having united with the water, and aſſumed an elaſtic form. Hence the aphoriſm already mentioned ſtands amply confirmed by experience, *that caloric is not capable of raiſing the temperature of bodies, however their form be changed by its combination with ſuch bodies.*

The late experiments made by Dr. CRAWFORD have ſatisfactorily taught us, what quantity or proportion of caloric is requiſite to unite with boiling water, in order to change it into a ſtate of vapour. This philoſopher inquired into the capacity which iron has of uniting with caloric, and found, that the proportion ſubſiſting between iron and water, in this reſpect, was eight times leſs in the iron than in the water. He employed, for inſtance, eight parts of iron filings, heated them to 300° of Fahrenheit, and poured to theſe *eight* parts of iron, *one* part of water, heated to 212°. He obſerved that the water was inſtantly converted into vapour, but the temperature of the vapour, as well as that of the mixture, amounted to 212° only; conſequently the iron had been deprived of 88° of caloric by means of the water; and theſe ought to have communicated to the water a temperature equal to 300°; but the temperature of the vapour remained equal to 212°, and hence we muſt conclude, that the 88° of caloric had actually been fixed by the water, and transformed it into a fluid vapour.

From what has been now advanced, it may be clearly perceived, that pure water is a body perfectly well adapted for the chemical analyſis of organic ſubſtances, in order to prevent that degree of heat by which their organic ſtructure might be deſtroyed, or at leaſt decompoſed and reduced to the more remote conſtituent parts; as it cannot be raiſed to a higher temperature than that of 212°, in which vegetable ſubſtances ſuffer no material change in their elementary conſtituents. As, farther, pure water is a very proper menſtruum for the greateſt number of thoſe formative principles which are diſcoverable in vegeto-organic bodies, it is eminently qualified to ſeparate them from other inſoluble ſubſtances, and to exhibit them in a ſtate, which enables us to proſecute the analytical method of examining their component parts with ultimate ſucceſs.

(To be continued in our next.)

